# Evaluation of the cobas SARS-CoV-2 & Influenza A/B v2 assay for detecting SARS-CoV-2 and influenza A and B viruses in nasopharyngeal swab specimens

**DOI:** 10.1128/spectrum.01111-25

**Published:** 2025-07-15

**Authors:** Eunjung Jeong, Minhee Kang, Hyeonseek Park, Ji-Youn Kim, Sun Ae Yun, Yun Young Cho, Jae-Hoon Ko, Tae Yeul Kim, Hee Jae Huh, Nam Yong Lee

**Affiliations:** 1 Biomedical Engineering Research Center, Smart Healthcare Research Institute, Samsung Medical Center 36626, Gangnam-gu, Seoul, Republic of Korea; 2 Research Institute for Future Medicine, Samsung Medical Center 36626, Gangnam-gu, Seoul, Republic of Korea; 3 Division of Infectious Diseases, Department of Medicine, Samsung Medical Center, Sungkyunkwan University School of Medicine 36626, Gangnam-gu, Seoul, Republic of Korea; 4 Department of Laboratory Medicine and Genetics, Samsung Medical Center, Sungkyunkwan University School of Medicine 36626, Gangnam-gu, Seoul, Republic of Korea; 5 Department of Medical Device Management and Research, Samsung Advanced Institute for Health Sciences & Technology, Sungkyunkwan University 35017, Gangnam-gu, Seoul, South Korea; Indiana University School of Medicine, Indianapolis, Indiana, USA

**Keywords:** SARS-CoV-2, influenza, cobas assay, Allplex assay

## Abstract

Accurate and rapid diagnostic assays that simultaneously detect severe acute respiratory syndrome coronavirus 2 (SARS-CoV-2) and influenza A/B are crucial for effective clinical management and infection control. In the present study, we evaluated the cobas SARS-CoV-2 & Influenza A/B v2 assay (Roche Diagnostics, Basel, Switzerland) using the Roche cobas 5800 system, comparing it to the Allplex SARS-CoV-2/FluA/FluB/RSV assay (Seegene, Seoul, Republic of Korea). A total of 871 nasopharyngeal swab specimens, including 164 SARS-CoV-2-positive, 76 influenza A-positive, 77 influenza B-positive, and 554 negative specimens, were tested using the cobas and Allplex assays in parallel. The positive percent agreement and negative percent agreement between the cobas and Allplex assays were 100% and 98.3% for SARS-CoV-2, 100% and 99.1% for influenza A, and 100% and 99.3% for influenza B, respectively. Cohen’s kappa values ranged from 0.95 to 0.96, indicating almost perfect agreement. The limits of detection for SARS-CoV-2 E and open reading frame 1ab genes, influenza A, and influenza B were 25.3, 12.4, 16.1, and 11.0 copies/mL, respectively. No cross-reactivity was observed with 29 non-target respiratory pathogens. These findings demonstrate that the cobas assay reliably detects SARS-CoV-2 and influenza A/B with high sensitivity and specificity. Notably, this assay enables high-throughput automated workflows, facilitating timely decision-making during SARS-CoV-2 and influenza cocirculation.

**IMPORTANCE:**

Accurate and rapid diagnostic assays that simultaneously detect severe acute respiratory syndrome coronavirus 2 (SARS-CoV-2) and influenza A/B are essential for effective clinical management and infection control. This study demonstrates that the cobas SARS-CoV-2 & Influenza A/B v2 assay reliably detects SARS-CoV-2 and influenza A/B with high sensitivity and specificity. Designed for use on the cobas 5800/6800/8800 systems, this assay supports high-throughput automated workflows, enabling timely decision-making during periods of high testing demand, such as the cocirculation of SARS-CoV-2 and influenza.

## INTRODUCTION

Accurate and timely detection of respiratory viruses, such as severe acute respiratory syndrome coronavirus 2 (SARS-CoV-2) and influenza A/B, is critical for effective clinical decision-making and public health management (1–3). These infections share overlapping symptoms, including fever, cough, and respiratory distress, which complicate diagnosis, especially during periods of viral co-circulation (4–6). Currently, molecular diagnostic assays are considered the reference standard for detecting and differentiating respiratory viruses (7, 8). However, many existing assays face limitations, including low throughput, extended turnaround times, and performance variability (9, 10). These challenges hinder their use, especially during periods of high diagnostic demand, such as seasonal influenza outbreaks and global pandemics.

 The cobas SARS-CoV-2 & Influenza A/B v2 assay (Roche Diagnostics, Basel, Switzerland) is a real-time reverse transcription polymerase chain reaction (rRT-PCR) test that recently received Emergency Use Authorization (EUA) from the U.S. Food and Drug Administration (FDA). This assay amplifies two targets for SARS-CoV-2: the envelope (E) gene and the open reading frame 1ab (ORF1ab) region. For influenza A, two genomic regions are targeted—the region encoding matrix proteins 1 and 2 (M1/M2) and the gene encoding polymerase basic protein 2. In contrast, influenza B is detected by amplifying a single genomic region encoding the nuclear export protein and nonstructural protein 1. Designed for use on the cobas 5800, 6800, and 8800 systems, this assay supports high-throughput automated workflows, enabling clinical laboratories to meet high diagnostic demand, especially during periods such as seasonal influenza outbreaks and global pandemics. Compared to its predecessor, the cobas SARS-CoV-2 & Influenza A/B v1 assay, the v2 assay offers enhanced analytical sensitivity, achieving lower limits of detection (LoD) across all target viruses (11). While the performance of the v1 assay has been evaluated in several studies (9, 12), limited data on the performance of the v2 assay are available. Moreover, the US FDA EUA for the v2 assay applies only to the cobas 6800 and 8800 systems, and its performance on the cobas 5800 system has not yet been established. This study assessed the diagnostic performance of the v2 assay, hereafter referred to as the cobas assay, using the cobas 5800 system and compared it to the Allplex SARS-CoV-2/FluA/FluB/RSV assay (Allplex; Seegene, Seoul, Republic of Korea). The Allplex assay was selected as a comparator due to its widespread use in clinical laboratories and proven diagnostic accuracy in previous studies (13–15).

## MATERIALS AND METHODS

### Clinical specimens

A total of 871 nasopharyngeal swab specimens, including 164 SARS-CoV-2-positive, 76 influenza A-positive, 77 influenza B-positive, and 554 negative specimens, tested by routine clinical testing between January 2016 and September 2024, were included in this retrospective study. SARS-CoV-2 positivity was determined using the PowerChek SARS-CoV-2 Real-time PCR Kit (Kogene Biotech, Seoul, Republic of Korea), while influenza A/B positivity was determined using the AdvanSure RV-Plus real-time RT-PCR assay (Invitros, Seoul, Republic of Korea), except for 11 influenza B-positive specimens identified by the BIOFIRE Respiratory 2.1plus Panel (bioMérieux, Marcy l’Étoile, France). Negative specimens were confirmed using the BIOFIRE Respiratory 2.1plus Panel. Positive specimens were selected based on the cycle threshold (Ct) values obtained from routine clinical testing, with Ct value distributions summarized in Table S1. All specimens were stored at −70°C until tested using the cobas and Allplex assays in parallel.

### Nucleic acid amplification tests

The cobas assay was performed according to the manufacturer’s instructions. Briefly, 600 µL of the nasopharyngeal swab specimen was transferred to a secondary tube and loaded onto the cobas 5800 system, where nucleic acid extraction, amplification, and detection were automatically performed.

 The Allplex assay, a multiplex rRT-PCR assay targeting the spike (S), RNA-dependent RNA polymerase (RdRp), and nucleocapsid (N) genes of SARS-CoV-2, the matrix (M) gene of influenza A, and the nonstructural (NS) gene of influenza B, was performed following the manufacturer’s instructions. Briefly, RNA was extracted using the MagNA Pure 96 system (Roche Diagnostics), and 10 µL of the extracted RNA was mixed with 10 µL of reaction master mix, yielding a final reaction volume of 20 µL. Multiplex rRT-PCR was conducted on the CFX96 Real-Time PCR Detection System (Bio-Rad Laboratories, Hercules, CA, USA) under the following cycling conditions: 50°C for 20 min, 95°C for 15 min, 3 cycles of 95°C for 10 s, 60°C for 40 s, and 72°C for 20 s, followed by 42 cycles of 95°C for 10 s, 60°C for 15 s, and 72°C for 10 s. A positive result was indicated by the presence of a well-defined exponential fluorescence curve crossing the threshold line at or before 38 cycles (40 cycles for RdRp).

 For specimens with discordant results between the cobas and Allplex assays, further analysis was performed using the PowerChek SARS-CoV-2 Influenza A & B Multiplex Real-Time PCR Kit (PowerChek; Kogene Biotech) according to the manufacturer’s instructions. This assay detects SARS-CoV-2 by targeting the E and ORF1ab genes, influenza A by targeting the M gene, and influenza B by targeting the nucleoprotein gene. In brief, RNA was extracted using the MagNA Pure 96 system. Then, 5 µL of extracted RNA was mixed with 15 µL of reaction master mix and 0.5 µL of internal control, yielding a final reaction volume of 20.5 µL. Multiplex rRT-PCR was performed on the CFX96 Real-Time PCR Detection System under the following cycling conditions: 50°C for 30 min, 95°C for 10 min, followed by 40 cycles of 95°C for 15 s and 60°C for 1 min. A positive result was indicated by the presence of a well-defined exponential fluorescence curve crossing the threshold line at or before 38 cycles.

 For all assays, specimens were considered inconclusive for SARS-CoV-2 if at least one, but not all, target genes were detected; such specimens required retesting.

### Analytical performance evaluation

The analytical sensitivity of the cobas assay was evaluated using the AMPLIRUN TOTAL SARS-CoV-2/FluA/FluB/RSV CONTROL (Vircell, Granada, Spain). The RNA standard was serially diluted in a pool of negative nasopharyngeal swab specimens and tested in 20 replicates per dilution level. The analytical specificity of the cobas assay was evaluated using a panel of 29 respiratory pathogens not targeted by the assay. Each pathogen was tested in duplicate at a clinically relevant concentration. Specifically, bacteria and yeasts were tested at ≥1 × 10^6^ CFU/mL or ≥1 × 10^6^ copies/mL, while viruses were tested at ≥1 × 10^5^ copies/mL.

### Statistical analysis

Two-by-two tables were used to evaluate the agreement between the cobas and Allplex assays. The positive percent agreement (PPA), negative percent agreement (NPA), Cohen’s kappa values, and two-sided 95% confidence intervals (CIs) were calculated. The correlation between the Ct values of the positive specimens in both assays was assessed using Pearson’s correlation coefficient. LoD calculations were performed using probit regression analysis. All statistical analyses were conducted using Excel (Microsoft, Redmond, WA, USA) and MedCalc Statistical Software version 23.0.6 (MedCalc Software Ltd., Ostend, Belgium).

## RESULTS

### Analytical performance

The LoDs for SARS-CoV-2 E and ORF1ab genes, influenza A, and influenza B were 25.3, 12.4, 16.1, and 11.0 copies/mL, respectively (Table 1). In the analytical specificity evaluation, no cross-reactivity was observed with 29 respiratory pathogens (Table 2).

**TABLE 1:** Analytical sensitivity evaluation results of the cobas assay

Concentration (copies/mL)	No. detected/No. of replicates			
	SARS-CoV-2		Influenza A	Influenza B
	E	ORF1ab		
100	20/20	20/20	20/20	20/20
50	20/20	20/20	20/20	20/20
25	18/20	20/20	20/20	20/20
10	14/20	18/20	12/20	17/20
5	10/20	11/20	5/20	18/20
2.5	6/20	10/20	3/20	13/20
1	2/20	6/20	0/20	5/20
Probit LoD (95% CI)	25.3 (19.1–39.9)	12.4 (9.2–21.6)	16.1 (12.7–24.6)	11.0 (8.0–21.3)

**TABLE 2:** Analytical specificity evaluation of the cobas assay^*a*^

Organism^*b*^	Source (code number)	Result
MERS-CoV	Vircell (MBC132)	Negative
Human coronavirus 229E	ATCC (VR-740D)	Negative
Human coronavirus OC43	Vircell (MBC135-R)	Negative
Human coronavirus NL63	Vircell (MBC142-R)	Negative
Human coronavirus HKU1	ATCC (VR-3262SD)	Negative
RSV type A	Vircell (MBC041)	Negative
Human parainfluenza virus 1	Vircell (MBC037)	Negative
Human parainfluenza virus 2	Vircell (MBC038)	Negative
Human parainfluenza virus 3	Vircell (MBC039)	Negative
Human parainfluenza virus 4	Vircell (MBC050)	Negative
Enterovirus D68	Vircell (MBC125)	Negative
Rhinovirus B14	Vircell (MBC091)	Negative
Human adenovirus 1	Vircell (MBC001)	Negative
Human metapneumovirus	KBPV (VR-87D)	Negative
Cytomegalovirus	NIBSC (09/162)	Negative
*Streptococcus pneumoniae*	ATCC (33400D-5)	Negative
*Haemophilus influenzae*	ATCC (51907D-5)	Negative
*Chlamydophila pneumoniae*	ATCC (53592D)	Negative
*Mycoplasma pneumoniae*	ATCC (15531D)	Negative
*Legionella pneumophila*	ATCC (33152D-5)	Negative
*Bordetella pertussis*	ATCC (9797D-5)	Negative
*Bordetella parapertussis*	ATCC (15311D-5)	Negative
*Staphylococcus aureus*	ATCC (29213)	Negative
*Staphylococcus epidermidis*	ATCC (12228)	Negative
*Streptococcus pyogenes*	ATCC (19615)	Negative
*Pseudomonas aeruginosa*	ATCC (27853)	Negative
*Neisseria meningitidis*	ATCC (13100)	Negative
*Escherichia coli*	ATCC (25922)	Negative
*Candida albicans*	ATCC (90028)	Negative

^*a*^ MERS-CoV, Middle East respiratory syndrome coronavirus; RSV, respiratory syncytial virus; ATCC, American Type Culture Collection; KBPV, Korea Bank for Pathogenic Viruses; and NIBSC, The National Institute for Biological Standards and Control.

^*b*^ Each organism was tested at clinically relevant concentrations: ≥1 × 10^6^ CFU/mL or ≥1 × 10^6^ copies/mL for bacteria and yeasts, and ≥1 × 10^5^ copies/mL for viruses.

### Clinical performance

**Fig 1:**
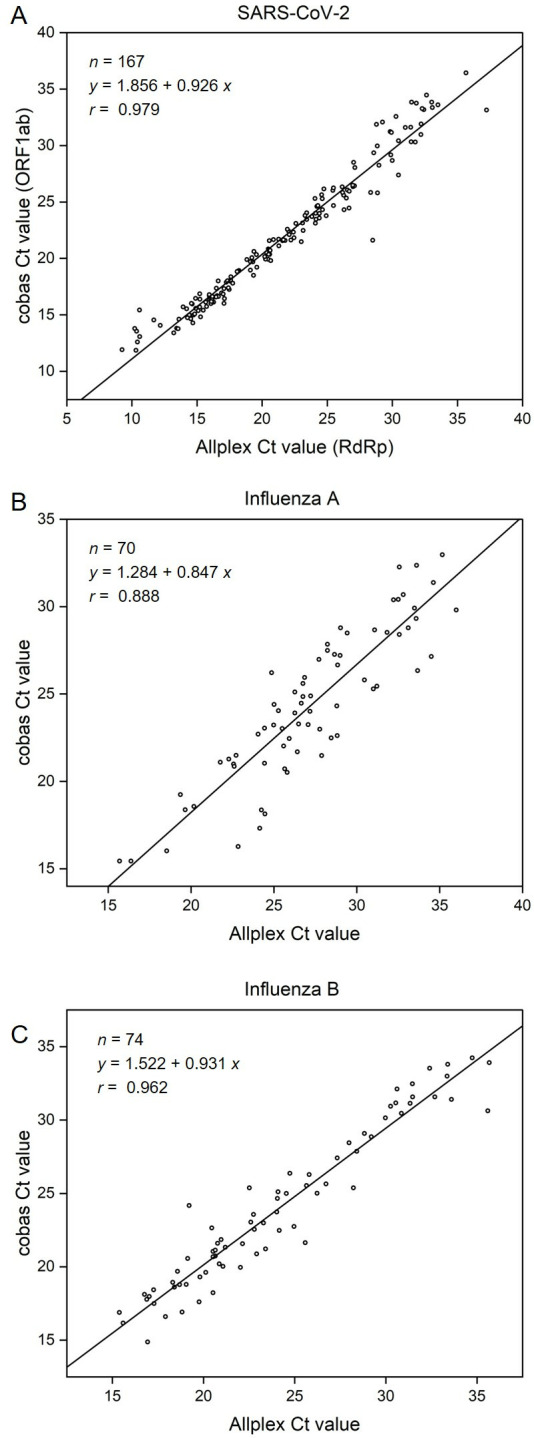
Correlation of Ct values for each target between the cobas and Allplex assays: (**A**) SARS-CoV-2, (**B**) influenza A, and (**C**) influenza B.

When analyzing 871 clinical specimens, the cobas and Allplex assays demonstrated high agreement for all three viruses, with a PPA of 100% and an NPA ranging from 98.3% to 99.3%. Cohen’s kappa values ranged from 0.95 to 0.96, indicating almost perfect agreement between the two assays (Table 3). Of note, two specimens tested positive for both SARS-CoV-2 and influenza A in the Allplex assay, with Ct values ranging from 19.2 to 25.0 for SARS-CoV-2 and 15.7–20.2 for influenza A. The cobas assay accurately detected both targets in these specimens, with Ct values ranging from 20.6 to 26.2 for SARS-CoV-2 and 15.4–18.6 for influenza A. Additionally, the Ct values obtained from the two assays exhibited a strong linear relationship for each viral target, with Pearson’s correlation coefficients ranging from 0.888 to 0.979 (Fig. 1).

**TABLE 3:** Comparison of the diagnostic performance of the cobas and Allplex assays

cobas result		Allplex result		PPA (95% CI)	NPA (95% CI)	Kappa value (95% CI)
		Positive	Negative			
SARS-CoV-2	Positive	167	12^*a*^	100% (97.8–100)	98.3% (97.1–99.0)	0.96 (0.93–0.98)
	Negative	0	692			
Influenza A	Positive	70	7	100% (94.9–100)	99.1% (98.2–99.6)	0.95 (0.91–0.99)
	Negative	0	794			
Influenza B	Positive	74	6	100% (95.1–100)	99.3% (98.4–99.7)	0.96 (0.92–0.99)
	Negative	0	791			

^*a*^ In the cobas assay, two specimens repeatedly yielded an inconclusive result for SARS-CoV-2. To calculate the agreement, these specimens were considered positive for SARS-CoV-2.

### Analysis of discordant results

Detailed results of the 25 specimens with discordant results are presented in Table 4. Among the 12 specimens that tested positive (*n* = 10) or inconclusive (*n* = 2) for SARS-CoV-2 in the cobas assay, all were negative in the Allplex assay. When further analyzed using the PowerChek assay, one specimen tested positive, two were inconclusive, and the remaining specimens tested negative for SARS-CoV-2. For influenza A and B, 13 specimens showed discordant results. Seven specimens were positive for influenza A in the cobas assay but negative in the Allplex assay. Of these, six yielded positive results for influenza A when tested with the PowerChek assay, while one yielded a negative result. Six specimens were positive for influenza B in the cobas assay but negative in the Allplex assay. Of these, three yielded positive results for influenza B when tested with the PowerChek assay, while three yielded negative results. All 25 specimens with discordant results had high Ct values for the corresponding targets (SARS-CoV-2: E gene, 34.1−36.9; ORF1ab gene, 34.6−36.8; influenza A: 28.6−36.0; and influenza B: 33.6−36.6), suggesting a low viral load in the specimens.

**TABLE 4:** Details of discordant results between the cobas and Allplex assays^*c*^

Specimen no.	Comparative testing		Discordant testing
	cobas assay	Allplex assay	PowerChek assay
	Detected target (Ct value^*a*^)	Detected target (Ct value^*a*^)	Detected target (Ct value^*a*^)
Specimens with discordant results for SARS-CoV-2 (*n* = 12)			
187	SARS-CoV-2 (35.2, 36.6)	ND	ND
199	SARS-CoV-2 (36.4, 36.2)	ND	ND
692	SARS-CoV-2 (36.1, 36.8)	ND	ND
727	SARS-CoV-2 (36.9, 35.2)	ND	ND
442	SARS-CoV-2 (34.1, 36.6)	ND	ND
775	SARS-CoV-2 (36.0, 36.7)	ND	SARS-CoV-2 (35.3, ND)^*b*^
806	SARS-CoV-2 (34.7, 35.0)	ND	SARS-CoV-2 (36.2, ND)^*b*^
737	SARS-CoV-2 (36.0, 34.6)	ND	SARS-CoV-2 (33.8, 37.7)
746	SARS-CoV-2 (36.6, 36.4), influenza A (23.0)	Influenza A (25.5)	Influenza A (23.6)
704	SARS-CoV-2 (36.0, 36.3), influenza B (33.9)	Influenza B (35.7)	Influenza B (34.2)
709	SARS-CoV-2 (35.7, ND)^*b*^	ND	ND
722	SARS-CoV-2 (ND, 36.8)^*b*^	ND	ND
Specimens with discordant results for influenza A (*n* = 7)			
46	Influenza A (30.8)	ND	Influenza A (32.9)
228	Influenza A (32.4)	ND	Influenza A (33.4)
372	Influenza A (33.8)	ND	Influenza A (34.6)
663	Influenza A (32.4)	ND	Influenza A (34.4)
687	Influenza A (34.1)	ND	Influenza A (35.3)
698	Influenza A (28.6)	ND	Influenza A (34.9)
507	Influenza A (36.0), influenza B (31.1)	Influenza B (31.3)	SARS-CoV-2 (35.2, 37.8), influenza B (31.8)
Specimens with discordant results for influenza B (*n* = 6)			
60	Influenza B (34.3)	ND	Influenza B (32.1)
615	Influenza B (34.1)	ND	Influenza B (34.5)
650	Influenza B (33.6)	ND	Influenza B (32.9)
678	Influenza B (36.6)	ND	ND
647	SARS-CoV-2 (24.6, 24.0), Influenza B (36.5)	SARS-CoV-2 (23.2, 24.4, 26.2)	SARS-CoV-2 (23.4, 26.4)
867	SARS-CoV-2 (26.4, 26.3), Influenza B (36.0)	SARS-CoV-2 (24.1, 25.5, 27.4)	SARS-CoV-2 (24.3, 28.0)

^*a*^ For SARS-CoV-2, Ct values correspond to target genes in the following order: cobas (E, ORF1ab), Allplex (S, RdRp, N), and PowerChek assays (E, ORF1ab).

^*b*^ These specimens were considered inconclusive for SARS-CoV-2, as only a single SARS-CoV-2 gene showed positive amplification, despite repeat testing.

^*c*^ ND, not detected.

## DISCUSSION

In this study, we evaluated the diagnostic performance of the cobas assay, a high-throughput automated molecular test for detecting SARS-CoV-2 and influenza A/B, and compared it with the Allplex assay, a widely used commercial rRT-PCR test. The cobas assay demonstrated high agreement with the Allplex assay for all target viruses, with a PPA of 100% and an NPA exceeding 98.3%. Furthermore, the cobas assay exhibited excellent analytical sensitivity and specificity. These findings suggest that the cobas assay is highly sensitive and accurate for detecting SARS-CoV-2 and influenza A/B. Given its ability to support high-throughput automated workflows, the cobas assay can serve as a valuable diagnostic tool for detecting SARS-CoV-2 and influenza A/B, particularly during periods of soaring testing demand.

 Despite the overall high agreement, 25 specimens with discordant results between the cobas and Allplex assays were observed. All these specimens were positive for the specific targets in the cobas assay but negative in the Allplex assay, possibly due to the superior analytical sensitivity of the cobas assay compared to the Allplex assay. We demonstrated that the cobas assay had excellent sensitivity, with LoDs of ≤25.3 copies/mL for all targets. These LoDs were much lower than those of the Allplex assay, as reported in a previous study (283.5–1,649.6 copies/mL for SARS-CoV-2, 4,917.3 copies/mL for influenza A, and 248.9 copies/mL for influenza B) (14). We attempted to confirm the presence of target viruses in specimens with discrepant results using the PowerChek assay; however, many of these specimens tested negative. Given the higher LoDs of the PowerChek assay (212.1–402.3 copies/mL for SARS-CoV-2, 5,661.8 copies/mL for influenza A, and 88.8 copies/mL for influenza B) compared to the cobas assay (14), the negative results from the PowerChek assay were likely false negatives. This suggests that the cobas assay offers a significant advantage in detecting low viral loads of SARS-CoV-2, influenza A, and influenza B that may not be detectable by other commercial molecular assays, such as the Allplex and PowerChek assays. Nevertheless, given the high Ct values observed in discordant specimens, the possibility of false-positive results with the cobas assay cannot be excluded. Further studies using highly sensitive molecular methods are warranted to resolve these discrepancies.

 One major advantage of the cobas assay is its ability to support high-throughput automated workflows. Designed for use on fully automated platforms—the cobas 5800, 6800, and 8800 systems—where sample preparation, nucleic acid extraction, amplification, and result reporting are automatically performed, the cobas assay minimizes manual handling and reduces the potential for human error (16–18). Commercial molecular assays, such as the BIOFIRE Respiratory 2.1 Panel (bioMérieux) and the Xpert Xpress SARS-CoV-2/Flu/RSV assay (Cepheid, Sunnyvale, CA, USA), are also designed for use on fully automated platforms (19–21). However, these assays may not be suitable for high-volume laboratories due to their limited testing capacity (22). In contrast, the cobas assay is well suited for high-volume laboratories due to its capacity to process a large number of specimens simultaneously. One disadvantage of the cobas assay, compared to widely used commercial molecular assays such as the Allplex assay, BIOFIRE Respiratory 2.1 Panel, and Xpert Xpress SARS-CoV-2/Flu/RSV assay, is its limited target coverage, particularly the absence of RSV detection. To address this, Roche has recently launched the cobas Respiratory flex test, which enables simultaneous detection of 12 respiratory viruses—including SARS-CoV-2, influenza A/B, and RSV—on the cobas 5800, 6800, and 8800 systems.

 One key limitation of this study is the use of archived specimens. The freeze-thaw process and long-term storage may have caused degradation of some viral RNA, potentially interfering with the accurate assessment of the cobas assay’s diagnostic performance. Additionally, the single-center design of the study limits its generalizability, highlighting the need for further evaluation across multiple testing sites. Finally, our study did not include positive specimens with Ct values > 35 due to the lack of such archived samples, which may represent a major limitation. Given the excellent analytical sensitivity of the cobas assay, it is expected to perform well in detecting low concentrations of target viruses; however, this should be further evaluated in future studies that include a sufficient number of positive specimens with Ct values > 35.

 In conclusion, our findings demonstrate that the cobas assay reliably detects SARS-CoV-2 and influenza A/B with high sensitivity and specificity. Moreover, its ability to support high-throughput automated workflows facilitates timely decision-making, particularly during periods of SARS-CoV-2 and influenza cocirculation.

## SUPPLEMENTAL MATERIAL

Table S1 spectrum.01111-25-s0001.docx Distribution of Ct values among selected positive specimens from routine clinical testing.

ASM does not own the copyrights to Supplemental Material that may be linked to, or accessed through, an article. The authors have granted ASM a non-exclusive, world-wide license to publish the Supplemental Material files. Please contact the corresponding author directly for reuse.
