# Supplementary material for: Evaluation of the cobas SARS-CoV-2 & Influenza A/B v2 assay for detecting SARS-CoV-2 and influenza A and B viruses in nasopharyngeal swab specimens
Source: Microbiol Spectr. 2025 Jul 15;13(8):e01111-25. doi: 10.1128/spectrum.01111-25 (PMC12323350; doi:10.1128/spectrum.01111-25)
Supplement: Table S1 — Distribution of Ct values among selected positive specimens from routine clinical testing. [file spectrum.01111-25-s0001.docx]

Table S1. Distribution of Ct values among selected positive specimens from routine clinical testing

|  | SARS-CoV-2 | | Influenza virus | |
| --- | --- | --- | --- | --- |
| Ct range | E | ORF1ab | Influenza A | Influenza B |
| <20 | 68 | 71 | 15 | 19 |
| 20–25 | 39 | 42 | 34 | 16 |
| 25–30 | 34 | 28 | 20 | 19 |
| 30–35 | 23 | 23 | 7 | 12 |
| Total no. | 164 | 164 | 76 | 66^a^ |

^a^ Eleven influenza B-positive specimens were confirmed using the BIOFIRE Respiratory 2.1plus Panel, which does not provide Ct values.
